# A revised Holocene coral sea-level database from the Florida reef tract, USA

**DOI:** 10.7717/peerj.8350

**Published:** 2020-01-20

**Authors:** Anastasios Stathakopoulos, Bernhard M. Riegl, Lauren T. Toth

**Affiliations:** 1 Halmos College of Natural Sciences and Oceanography, Guy Harvey Oceanographic Center, Nova Southeastern University, Dania, FL, United States of America; 2 St. Petersburg Coastal and Marine Science Center, U.S. Geological Survey, St. Petersburg, FL, United States of America

**Keywords:** Holocene, Sea level, Coral reefs, *Acropora palmata*, Florida reef tract, Western atlantic

## Abstract

The coral reefs and mangrove habitats of the south Florida region have long been used in sea-level studies for the western Atlantic because of their broad geographic extent and composition of sea-level tracking biota. The data from this region have been used to support several very different Holocene sea-level reconstructions (SLRs) over the years. However, many of these SLRs did not incorporate all available coral-based data, in part because detailed characterizations necessary for inclusion into sea-level databases were lacking. Here, we present an updated database comprised of 303 coral samples from published sources that we extensively characterized for the first time. The data were carefully screened by evaluating and ranking the visual taphonomic characteristics of every dated sample within the database, which resulted in the identification of 134 high-quality coral samples for consideration as suitable sea-level indicators. We show that our database largely agrees with the most recent SLR for south Florida over the last ∼7,000 years; however, the early Holocene remains poorly characterized because there are few high-quality data spanning this period. Suggestions to refine future Holocene SLRs in the region are provided including filling spatial and temporal data gaps of coral samples, particularly from the early Holocene, as well as constructing a more robust peat database to better constrain sea-level variability during the middle to late Holocene. Our database and taphonomic-ranking protocol provide a framework for researchers to evaluate data-selection criteria depending on the robustness of their sea-level models.

## Introduction

The societal implications of projected future sea-level rise are of great concern. Because over a quarter of the world’s population resides near the coast (Kummu et al., 2016), large-scale (and particularly, rapid) coastal flooding could result in high losses of lives, property, and a cascade of severe socio-economic consequences. As humankind contemplates how to cope with future sea-level rise, it has become increasingly clear that a better understanding of the natural variability in the magnitudes and rates of sea-level rise in the past is needed to more accurately predict future scenarios (Kopp et al., 2009; Dutton & Lambeck, 2012; Dutton et al., 2015). In this regard, examinations of Holocene (last 11,700 years) sea-level rise could provide critical and useful baseline information for researchers to parameterize predictive models and to generate realistic future projections (Horton et al., 2018).

At present, relative sea level can be defined as the local elevation of the ocean’s surface referenced to a datum such as the land surface of the ocean floor (Shennan, 1986; Van de Plassche, 1986; Khan et al., 2017). Sea-level reconstructions prior to instrumental records are dependent on proxy indicators that have a known and quantifiable relationship to sea level (Shennan, 2015). By measuring the elevation and radiometric age of sea-level indicators and compiling the data into time-elevation plots, sea-level reconstructions (SLRs, commonly referred to as “sea-level curves”) can be developed. Scleractinian (reef-building) corals, mangrove peats, lithified beach/dune ridges and/or combinations thereof are the most commonly-used indicators of relative sea level in the tropics and subtropics. Each of these proxies has inherent advantages and disadvantages for interpretation, and care must be taken when analyzing data from each of these sources. For example, scleractinian corals grow subtidally across depths that range from several meters to tens of meters (Hibbert et al., 2016), making the relationship of various taxa to sea level difficult to pinpoint. Recent work by Hibbert et al. (2016) and Hibbert et al. (2018) showed that modeling the modern distributions of individual coral taxa within particular regions can help to constrain coral indicative ranges (i.e., the depth range occupied by the coral), potentially allowing them to be used as quantitative sea-level indicators; however, in the western Atlantic, in situ, monospecific framework of *Acropora palmata* is the primary quantitative coral sea-level indicator that has been used in SLRs to date because of this species’ characteristic growth morphology and formation of monospecific framework in shallow (typically <5 m) water (Lighty, Macintyre & Stuckenrath, 1982). This indicator is not without limitations in the geologic record, however, as studies have shown that the internal composition of some shallow Caribbean fringing reefs dominated by acroporids are comprised of storm-generated clasts with only minor amounts of in situ framework (Blanchon, Jones & Kalbfleisch, 1997; Perry, 2001; Blanchon & Perry, 2004; Blanchon et al., 2017). Furthermore, undifferentiated *A. palmata* samples (i.e., those that are not clearly from reef-crest environments) can possess vertical uncertainties of up to 10–15 m in their indicative range, which decreases the precision of SLRs if they are included (Blanchon & Perry, 2004; Blanchon, 2005). Studies of Caribbean Holocene reefs over the last several decades have demonstrated that reefs generally consist of varying combinations of coral skeletons and other calcifying organisms, void space, and sand. For example, Hubbard, Burke & Gill (1998) examined the composition of several eastern Caribbean reefs and found that they were comprised of only 30% of intact corals, of which less than half were clearly in situ. Similarly, a study of Campeche Bank reefs by Blanchon & Perry (2004) reported that cores from shallow reef-crest and reef-front zones contained 30–40% of in situ corals. Other studies have reported the presence of subaerial boulder ramparts and rubble cays principally composed of *A. palmata* clasts on shorelines adjacent to reefs (Williams et al., 1999; Morton et al., 2008) and that cores from the ramparts can be difficult to distinguish from reef crest facies (Blanchon & Perry, 2004). Indeed, such studies have shown that identifying in situ corals from the geologic record is possible, but challenging (Hubbard, Burke & Gill, 1998), and they highlight the importance of distinguishing and screening out allochthonous samples when creating SLRs (Blanchon & Perry, 2004).

Similarly, the use of mangrove peats as sea-level indicators requires the consideration of several caveats. Mangrove peat accumulation occurs in the upper half of the intertidal zone (mean tide level to highest astronomical tide, Twilley, 1985; Woodroffe et al., 2016; Khan et al., 2017); however, *Rhizophora mangle* (red mangrove) peats form closer to mean tide level than the slightly more elevated *Avicennia germinans* (black mangrove Toscano & Macintyre, 2003; Khan et al., 2017), which makes the former taxon a more precise sea-level indicator. Additionally, radiometric dating of peats could be affected by factors such as compaction or reworking of deposits, incorporation of younger roots or older carbon, and bacterial contamination of unrefrigerated samples (Törnqvist et al., 2004; Gischler, 2006; Toscano, Gonzalez & Whelan, 2018). Furthermore, although mangrove peats are more precise sea-level indicators compared to corals, multi-millennial records and early Holocene-aged samples of peats are scarce in many locations, including south Florida. Temporal gaps in the records of coral-based indicators can also occur as a result of biases in sampling depths (i.e., exclusion of the deepest and shallowest reef zones; e.g., Shinn, 1980) and hiatuses in reef development. For example, recent studies by Stathakopoulos & Riegl (2015) and Toth et al. (2018) showed that most reefs throughout the Florida reef tract stopped accreting or were accreting at negligible rates by the late Holocene. Combining coral and peat data in multi-proxy reconstructions can help alleviate the tradeoffs between the two indicators (see Toscano & Macintyre, 2003; Milne & Peros, 2013; Khan et al., 2017).

The nature of Holocene sea-level rise in the western Atlantic has been contested among researchers, resulting in considerable disagreements about the rates of rise during particular periods (Toscano & Macintyre, 2003; Gischler, 2006; Toscano & Macintyre, 2006), the occurrence of rapid sea-level “jumps” (Blanchon, Jones & Ford, 2002; Blanchon, 2005) of several meters over a few hundred years caused by ice-sheet collapse (Blanchon & Shaw, 1995), and the potential for oscillations between sea-level highstands and lowstands during the middle–late Holocene (Balsillie & Donoghue, 2004). Interestingly, the same suite of data from south Florida were used as evidence both for, and against, putative jumps in sea level during the early Holocene (Toscano & Macintyre, 2003; Blanchon, 2005; Toscano & Macintyre, 2005). In addition, Brock et al. (2008) and Brock et al. (2010) proposed that high-frequency sea-level oscillations during the middle–late Holocene depicted in the Balsillie & Donoghue (2004) SLR for the northern Gulf of Mexico could explain observed patterns of reef geomorphology from two locations in south Florida.

An earlier sea-level study by Lighty, Macintyre & Stuckenrath (1982) provides important background context for SLRs in south Florida since it was one of the primary datasets utilized in updated SLRs by subsequent researchers. The Lighty, Macintyre & Stuckenrath (1982) SLR was among the first regional studies to describe the general characteristics and magnitude of sea-level change over the Holocene by using *A. palmata* sea-level data from several western Atlantic sites, including Florida. Toscano & Macintyre (2003) utilized and expanded upon the dataset of Lighty, Macintyre & Stuckenrath (1982) by adding new *A. palmata* and mangrove peat sea-level data and applying radiocarbon corrections to produce an updated Caribbean-wide SLR. More recently, Milne & Peros (2013) and Khan et al. (2017) created location-specific SLRs using new data from several western Atlantic sites, including Florida. They also included much of the original data from Lighty, Macintyre & Stuckenrath (1982) and Toscano & Macintyre (2003) but applied geophysical and statistical models to interpret those data. It is important to note that none of the SLRs described above and analyzed herein performed similar detailed taphonomic characterizations to screen and incorporate only in situ coral samples, and instead, relied upon the original author’s descriptions of the samples rather than systematic screening approaches (see below and Methods section for further details).

Most SLRs using data from south Florida depict a relatively smooth rise in sea level to present in most of the western Atlantic following the last deglaciation (Milne, Long & Bassett, 2005; but see Blanchon, 2005) and suggest that the rate of relative sea-level rise was fastest during the early Holocene (∼11.7–8 ka), began to slow during the middle Holocene (∼8–4 ka), and sea level was within a few meters of its modern position by the late Holocene (∼4 ka–present). The timing of these changes is in general agreement with some studies of eustatic sea-level change following deglaciation (Wanless, Parkinson & Tedesco, 1994; Lambeck et al., 2014; Peltier, 2015); however, studies from other locations have documented rapid jumps in sea level of a few meters between ∼8.5–8.2 ka (Hijma & Cohen, 2010; Törnqvist & Hijma, 2012; Hijma & Cohen, 2019) and suggest that eustatic sea-level rise was not monotonic. The discrepancies among the aforementioned sea-level studies combined with the utilization of sea-level data largely based on an older, unverified coral database from south Florida by subsequent researchers highlight the need to evaluate the reliability of the data used in these SLRs.

Here, we expand upon the coral database of sea-level indicators used in previously published Holocene SLRs for south Florida by analyzing the extensive coral-reef core records collected from the region over approximately the last five decades. We compiled all relevant published information and then visually inspected and characterized the taphonomic indicators for every dated coral sample from the literature. This approach allowed us to make determinations about whether each sample was collected in situ (in growth position) or represented an allochthonous deposit, what depth-related reef zone the sample likely came from (e.g.,  Blanchon & Perry, 2004; Perry & Hepburn, 2008), and ultimately whether the sample is a useful indicator for SLRs. Our study, therefore, provides a test of the reliability of coral samples used in previously published SLRs by ranking and screening samples based on taphonomic and other qualities. We compare our updated sea-level database against the five most recent SLRs used for south Florida to evaluate how well our characterizations agree with these models and we identify when and where additional data are needed to refine future SLRs. Our detailed taphonomic characterizations and rigorous sample-screening protocol are the first of their kind for the region and provide data-quality indicators for presently available coral samples to evaluate previously published SLRs and to construct new SLRs for south Florida. This screening framework and the resulting identification of high-quality Holocene sea-level indicators from the region will allow researchers using these data to have higher confidence in their estimates of the rates and magnitudes of sea-level rise during an important warm interval in Earth’s recent past (Dutton et al., 2015). These methods could be further applied by researchers interested in developing or revising coral-based SLRs throughout the western Atlantic to increase the robustness and precision of their datasets and models.

## Methods

### Study area

**Figure 1:**
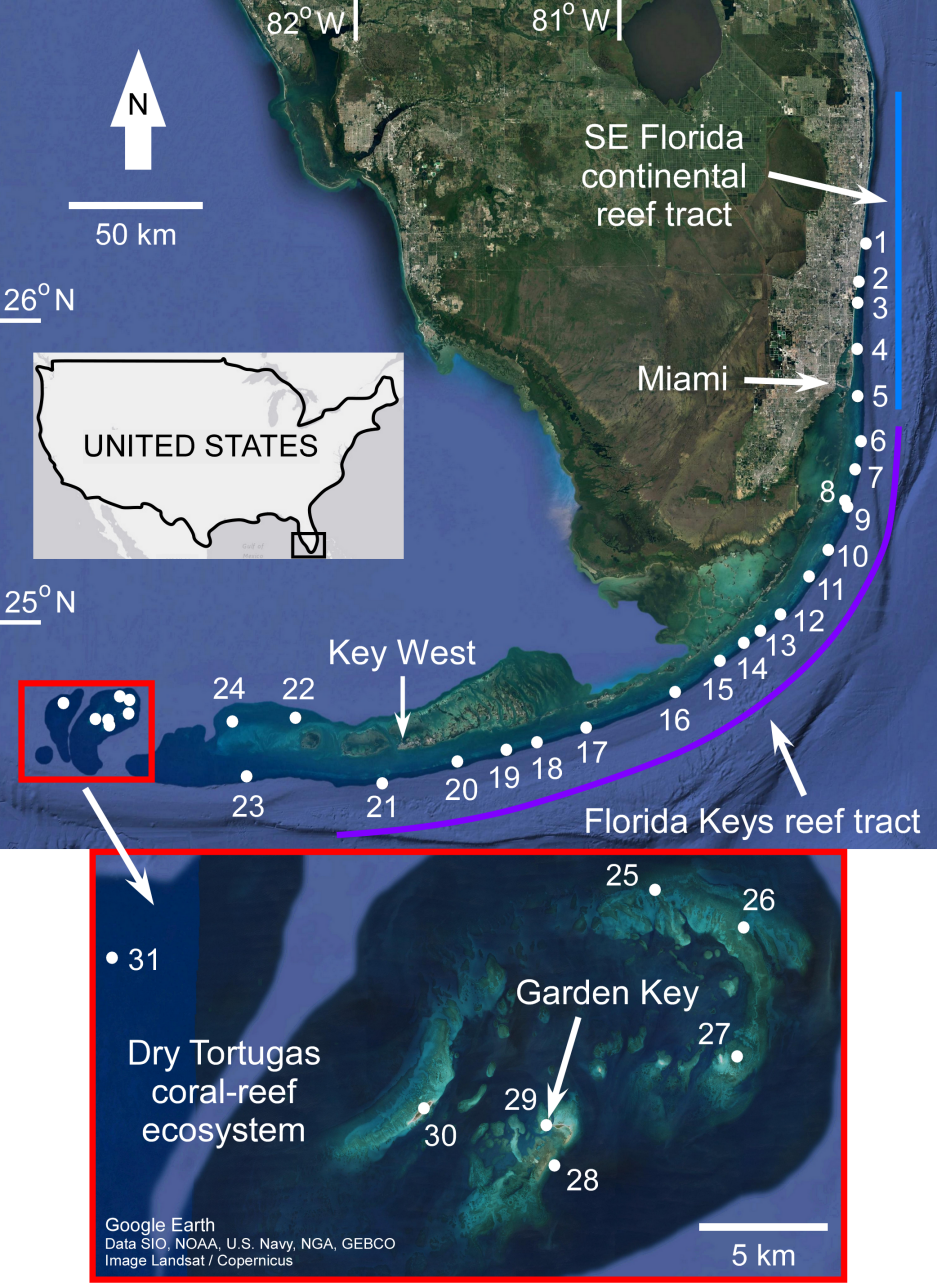
Map of coral sample locations from south Florida. The locations of coral samples comprising the sea-level database are depicted by *numbered white circles* (see Stathakopoulos & Toth, 2019 for site names). The extent of the three reef subregions are indicated by *colored lines.* The *light blue line* indicates the Southeast (SE) Florida continental reef tract, the *purple line* indicates the Florida Keys reef tract, and the *red box* (also inset) indicates the Dry Tortugas coral-reef ecosystem. Map from Google Earth, Data SIO, NOAA, U.S. Navy, NGA, GEBCO, Image Landsat/Copernicus.

The geomorphology of the Florida reef tract has been extensively reviewed in the literature (Lidz, Reich & Shinn, 2003; Mallinson et al., 2003; Banks et al., 2007; Brock et al., 2010; Shinn & Lidz, 2018; and references therein), and will only be briefly summarized here. The reef tract can be divided into three distinct subregional reef systems based on their unique accretion history, physical environment, and location on the shelf (Fig. 1). Together, they form a mostly continuous complex of coral-reef habitats ∼500 km in length that parallel the Atlantic shoreline of south Florida. In a north–southwest direction they are: the Southeast Florida continental reef tract (SFCRT), the Florida Keys reef tract (FKRT), and the Dry Tortugas coral-reef ecosystem (DTCRE, after Shinn et al., 1989; Banks et al., 2007; Brock et al., 2010). Historically, the Dry Tortugas reefs have been considered to be a part of the FKRT, but for this study it will be evaluated separately due to its unique oceanographic and geographic setting (see Toth et al., 2017a) and geomorphology (Brock et al., 2010). Note that samples from the Marquesas Islands were grouped with the DTCRE for the same reason. The term ‘Florida reef tract’ is used herein to refer to the combined extent of the three reef subregions situated on the south-Florida shelf.

### Sea-level data selection criteria

Holocene coral age and elevation data from the Florida reef tract were identified and extracted from relevant literature published prior to the end of the 2017 calendar year (sources and data are available at https://doi.org/10.5066/P98QFBJ3, Stathakopoulos & Toth, 2019). The coral samples were originally obtained utilizing various geological sampling methods (primarily rotary reef coring), and were analyzed using various radiometric dating techniques, calibrations, and elevation estimates. Data are scattered widely throughout the literature and through time, owing in part to the inherent technical difficulty of obtaining samples from submerged coral reefs, especially via core-drilling. Our database includes older data that were not incorporated into published SLRs, as well as recently published data that became available after the creation of those SLRs. In addition, important cataloging information pertaining to the samples and the radiometric-dating laboratories that processed them (e.g., elevation datums and/or *δ*^13^C values) were variably reported in the original studies. We sought out this information by personal communication with the original researchers and/or dating laboratories where possible. All relevant information (e.g., core names, radiometric age, coral taxon) from the original publications were noted in the database. The majority of the cores extracted from the Florida reef tract are presently stored in the core archive at the U.S. Geological Survey Coastal and Marine Science Center in St. Petersburg, Florida (USGS Core Archive; see https://doi.org/10.5066/F7319TR3; Reich et al., 2012). Descriptive core logs, photographs, and additional data are provided for these cores in Toth, Stathakopoulos & Kuffner (2018); https://doi.org/10.5066/F7NV9HJX and can be used in conjunction with the tabulated taphonomic- and core-descriptions from our database (Stathakopoulos & Toth, 2019) to further interpret the records provided therein.

### Calibration of age and elevation data

Samples in our database have been analyzed by Uranium-series (U-series) and radiocarbon (^14^C) dating methods, requiring both radiometric age-dating types to be calibrated to a common temporal framework for direct comparison. U-series ages were recalculated using the most recent decay constants reported by Cheng et al. (2013) for ^230^Th and ^234^U and by Jaffey et al. (1971) for ^238^U, with ages reported relative to 1950 CE. U-series data were screened for: open-system behavior indicated by *δ*^234^U_initial_ values outside of 137–157‰ and incorporation of detrital thorium indicated by ^232^Th values >2 ppb. In addition, we screened the ^238^U data for values outside of the typical ranges of 2,800–3,800 ppb for acroporid corals and 2,000–3,200 ppb for massive corals (Cross & Cross, 1983; Muhs et al., 2011) similar to Toth et al. (2017a). Samples that did not pass our screening procedure may have been diagenetically altered, which could lead to erroneous age calculations (see Scholz & Mangini, 2007; Stirling & Andersen, 2009; Dutton, 2015). Original U-series data, new recalculations, and data screening are provided in Table S1.

For radiocarbon ages, if no conventional ^14^C ages or *δ*^13^C values were originally reported then we corrected ^14^C ages to conventional ^14^C ages a *δ*^13^C value of 0 ± 4‰ (adapted with from Stuiver & Polach, 1977) using the Calib spreadsheet “d13ccorr.xls” (Stuiver, Reimer & Reimer, 2019). Conventional ^14^C ages were converted to calibrated calendar years using the CALIB 7.0.2 computer program accessible at http://calib.org/calib/ (Reimer & Reimer, 2001). The most recent calibration datasets, IntCal13 and Marine13 (Reimer et al., 2013), were used for peat and coral samples respectively. For marine samples, we applied the time-varying ΔR (local marine reservoir correction offset) values calculated by Toth et al. (2017a) and, if necessary, ΔR values were interpolated between the values reported at 5-year intervals (Toth et al., 2017b). According to Toth et al. (2017a), ΔR varied considerably during the Holocene between open ocean (DTCRE) and nearshore environments (SFCRT and FKRT) until ∼4 ka, with the former generally having greater ΔR values. In our database ΔR ranged from 11.43 to −110.58 years with an average of −34.23 years. Radiocarbon ages are reported in calibrated calendar years before present (cal BP) relative to 1950 CE as the central intercept of the calibration curve and are temporally consistent with absolute U-series ages. The shorthand notation for thousands of calibrated years before 1950 CE (ka) is used herein for both types of radiometric ages.

The quality of elevation estimates from published studies varies considerably. For example, reef-surface elevations (and therefore sample elevations) were often measured using SCUBA-diving depth gauges and the oldest studies often referenced a generic “water depth”, while others referenced elevation relative to mean sea level (MSL), or to a geodetic datum. Local tidal corrections were applied by some researchers using tidal datums for further elevation accuracy, however, they were not applied consistently across studies. In our database, we converted elevation data referenced to a geodetic datum to MSL using the nearest, local tidal datums available at http://www.tidesandcurrents.noaa.gov (accessed on 2018-9-1); however, the remainder of the data are presented as they were originally reported (unless noted otherwise Stathakopoulos & Toth, 2019), rounded to the nearest 0.05 m. All elevation data presented in our database, therefore, are referenced to MSL except those originally reported as a “water depth” because no corrections and conversions could be applied to those samples due to the lack of additional detailed information regarding the time and date of collection. We also estimated the total elevation uncertainty (±2*σ*) of each coral sample by calculating the root-sum-square of the individual uncertainties (±2*σ*) based on (1) establishing the sample’s elevation in the field (e.g., survey), (2) the method of sample acquisition (e.g., coring), and (3) sampling (e.g., subsampling and processing a section of core) after Hijma et al. (2015), Hibbert et al. (2018), and references therein. We applied a ±0.5 m (2*σ*) uncertainty for elevation determination based on the use of a digital depth gauge/dive computer or if unknown/not reported, ±0.15 m (2*σ*) uncertainty for coring methods using a rotary drill, and ±0.01 m (2*σ*) uncertainty for sampling cores and outcrops (see Table 3 of Hibbert et al., 2018). All uncertainty values and total sample elevation uncertainties for coral samples are provided in Stathakopoulos & Toth (2019).

### Characterization and screening of coral samples

Every dated sample identified from the literature was first cross-referenced with the USGS Core Archive to determine if the core material was physically available for analysis. If samples were not present in the archive, we solely relied on the information and interpretations provided in the original publication to populate the database (Stathakopoulos & Toth, 2019). Samples that were stored in the physical archive were visually inspected to identify taphonomic characteristics (following the guidelines of Blanchon & Perry, 2004; Perry & Hepburn, 2008; and references therein) that aided in determining whether the samples were collected in situ, and ultimately, whether they are suitable sea-level indicators. We note that 117 of the 257 available coral samples analyzed were from cores that were partially or fully slabbed in half and our visual analyses for the remainder of the samples were, therefore, made only on the external surfaces. The criteria we assessed to determine if coral samples were in situ included the presence of: basal attachment surfaces (which form when a coral encrusts the substrate it is growing on), normally oriented (i.e., vertical) corallites, and normally oriented geopetals (cavities filled with sediment). Due to the radial growth structure of *A. palmata* corals, normal corallite orientation for this species was determined by the distinctive asymmetrical growth of its corallites (Lighty, Macintyre & Stuckenrath, 1982) and the orientation of the growth axis of branches with angles >20° from the horizontal (Blanchon & Eisenhauer, 2001).

To increase the breadth of the database for future users and to provide greater context for the cores and samples, we also assessed several secondary criteria, which were only used to aid in the identification of former reef zones or facies (according to Blanchon & Perry, 2004). These secondary criteria included: the presence and thickness of intergrown vermetid worms, crustose coralline algae (CCA), and the benthic foraminifer *Homotrema rubrum* (i.e., intergrown encrusters), the character of submarine cements, and the dominant composition and orientation of bounding coral facies. We characterized the secondary criteria by first describing the intergrown encrusters and submarine cements for the dated sample, then we analyzed the 50 cm of core material above and below the dated sample to determine the dominant coral composition of that section (i.e., massive vs. *A. palmata* facies). Next, we described the primary and secondary taphonomic indicators of the 1-m section of core around the dated sample to better characterize the section that the sample was emplaced within. If this 1-m section was dominated by *A. palmata*, we determined the total continuous length of the *A. palmata* section throughout the rest of the core, and then further examined the taphonomic indicators and condition of all corals and/or clasts within the overall larger interval. The combination of this information was assessed to determine if we could identify reef facies signatures (i.e., reef crest, shallow reef front, subaerial rubble cay/ridge) within the core section and if the dated *A. palmata* sample(s) could be interpreted to be from a section of monospecific framework or a clast-dominated sedimentary unit. For intervals that we interpreted as reef crest facies, we assessed the percentage of *A. palmata* corals >5 cm in length with normally oriented corallites (as described above) within these sections and assumed values ≥50% to represent in place framework. All taphonomic characterizations are provided in Stathakopoulos & Toth (2019). Furthermore, any obvious discrepancies between our observations of the cores and the published literature were corrected and updated in the database (the most common discrepancies were mis-identification of coral species).

The overall characterization of the samples was used to rank each sample (from 0–3, with 0 being the highest rank) in terms of the quality and reliability of the sea-level data it could provide (Stathakopoulos & Toth, 2019). Samples were excluded outright (rank = 3) if one of the following criteria were met: (a) the core or sample was not available for observation and no other direct evidence (e.g., photographs or detailed field notes) of in situ characteristics were provided by the original researchers or described in the original publication, (b) the sample did not pass U-series screening (either performed in the original publication, or described above), and/or (c) the sample did not pass diagenetic screening if performed in the original publication (i.e., X-ray diffraction or scanning electron microscope analyses). Samples were also excluded (rank = 2) if they were emplaced within a predominant sand interval or an interval of no to very low recovery (<10%), if there was a significant age-reversal (i.e., outside of ±2*σ* age-range) between an adjacent dated sample, and/or the sample possessed inverted corallites and/or geopetals (indicating that the sample was not in situ). For samples that possessed ambiguous features (e.g., corallite orientation was indeterminate, but possessed a normal geopetal) or were otherwise not clearly definitive, a rank = 1 was assigned, whereas those that had clearly distinguished in situ characteristics (e.g., normal corallite orientation and no other ambiguous features) were assigned a rank = 0. We applied a conservative screening approach and only interpreted samples with a rank = 0 as being in situ specimens.

We note that potential differences between our observations of taphonomic characteristics herein and those by Blanchon & Perry (2004), which much of our criteria were based on, may be related to the size (diameter) of cores recovered. Their assessments were based on large-diameter cores (∼9 cm) that only penetrated a maximum of ∼1.6 m of Holocene reef framework and radiometric ages from their cores generally only spanned ∼1,000 years. All the reef cores examined from the USGS Core Archive are small-diameter (range from ∼3.5–6 cm), which is necessary for retrieving the entire section of Holocene reef framework in south Florida (generally ∼3 m thick, but up to ∼15 m); however, these cores yield data spanning several millennia. Whereas larger diameter cores are certainly ideal in particular applications (e.g., detailed examination of the immediate reef-subsurface), they are not designed to penetrate to depths that double-barrel diver-operated drilling systems can, which is why the latter are most-commonly utilized in reef-drilling studies (see Hubbard, 2011 for a review). An issue with coring studies in general is that cores only provide narrow, one-dimensional views of the reef interior that can exclude important taphonomic characteristics from the boundaries of observation (Hubbard, Burke & Gill, 1998; Blanchon & Perry, 2004; Hubbard, 2011); however, differences attributed to core-diameter size in the recovery of material with more readily recognizable taphonomic characteristics are difficult to account for because no other similar studies exist for direct comparison.

### Database evaluation against Holocene SLRs

We plotted all coral age-elevation data that passed our strictest screening criteria (i.e., rank = 0) against recent SLRs used in studies of the south Florida region which include: Toscano & Macintyre (2003), Balsillie & Donoghue (2004), Blanchon (2005), Milne & Peros (2013), and Khan et al. (2017). It should be noted that the Balsillie & Donoghue (2004) SLR was created for the Gulf of Mexico region but was used by Brock et al. (2008) and Brock et al. (2010) to infer sea-level and associated reef accretion processes in south Florida. In addition, the ‘Blanchon (2005) SLR’ (terminology used herein only for simplicity) was a comment on the assumptions of the Toscano & Macintyre (2003) SLR and was intended to be a reinterpretation of the data rather than a SLR. We evaluated the SLRs of the five aforementioned studies because they were constructed using data from south Florida or were utilized in discussions about sea level in the region. We accounted for the full vertical uncertainties of the coral data on our plots by combining the elevation uncertainties (±2*σ*, see ‘Methods’) and the coral taxa depth distributions at 95% confidence intervals (±2*σ*) using the methodology outlined in Hibbert et al. (2016) and Hibbert et al. (2018). A coral’s paleo water depth was therefore estimated using the corrected coral elevation and we used the root-sum-square of the elevation uncertainties as the negative uncertainty for the corrected coral elevation. The positive uncertainty of the corrected coral elevation included the root-sum-square of both the elevation uncertainties and the coral taxa depth distributions. For each coral taxon we used the depth distribution from the Florida regional depth distributions if reported by Hibbert et al. (2018), otherwise we used the Caribbean-wide distributions reported in Hibbert et al. (2018), or Hibbert et al. (2016) if not reported in the former. If no depth distribution was reported for a given coral taxon (e.g., *Stephanocoenia intersepta*) or if the coral was not identified to genus or species (e.g., “brain coral”) we assigned a maximum error value based on the most closely associated coral species. All the taxon-specific depth distributions for each sample and their full vertical uncertainties are provided in Stathakopoulos & Toth (2019). Whereas we display the coral uncertainties as symmetrical, Gaussian (2*σ*) distributions in our figure plots, we note that relative sea level would most likely be closer to the corrected coral positions (i.e., the coral markers in our figure plots) because most coral species distributions peak in the upper water column (Hibbert et al., 2018). In addition, the non-coral age-elevation data reported in Appendix 3 of Khan et al. (2017) were also plotted, along with their 2*σ* error values for “Compaction-adjusted RSL” and “Age”. For simplicity, horizontal error bars (i.e., 2*σ* age uncertainties) are only depicted for samples that have age-error ranges ≥250 years. We emphasize that no new SLR is produced in this study.

## Results

### Database metrics

**Figure 2:**
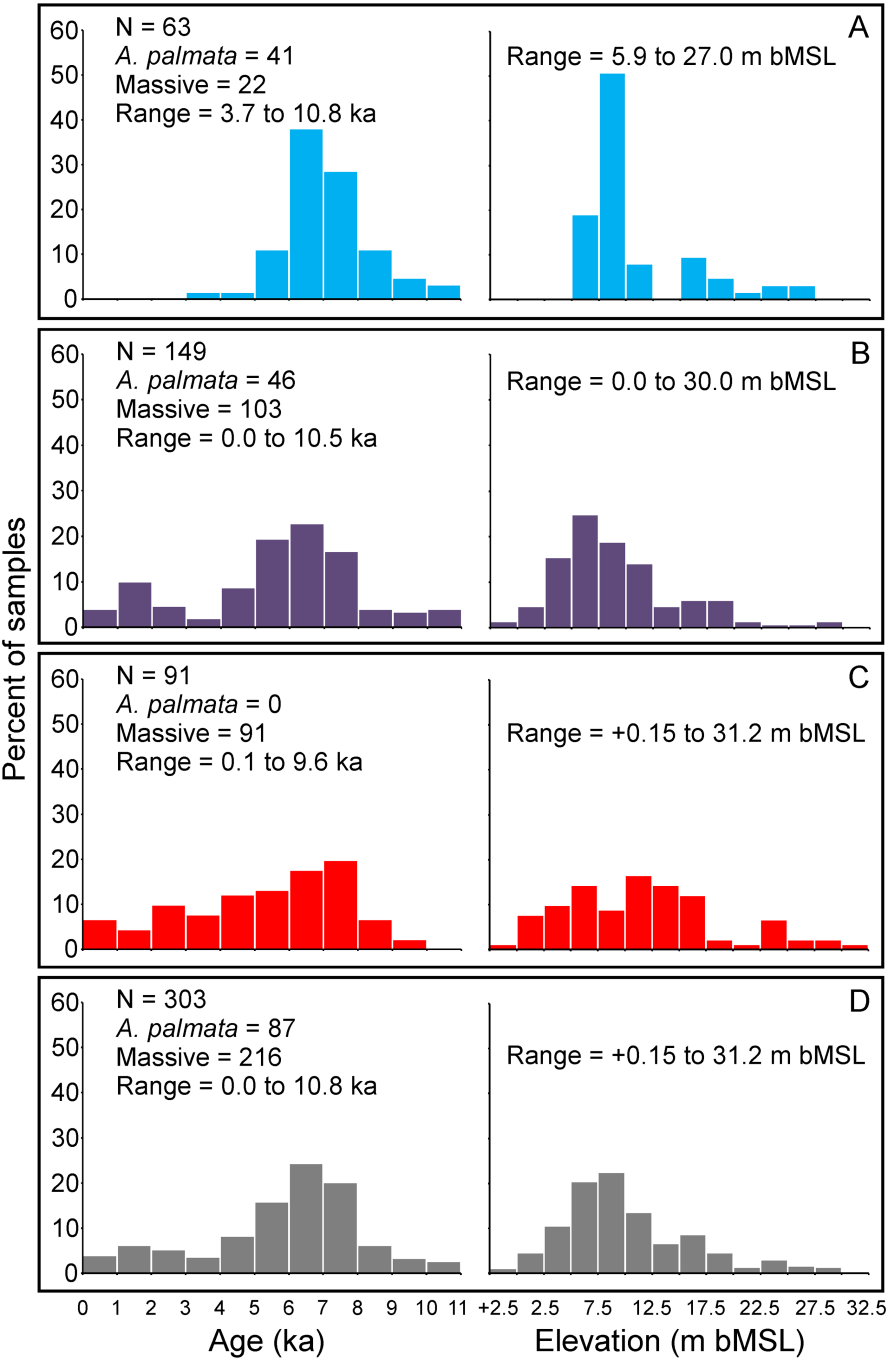
Summary histograms of all coral age- and elevation-data (*left and right sides of panels respectively*) comprising the Florida reef tract database. *Colors* indicate reef subregions as depicted in Fig. 1. Elevation is m below mean sea level (bMSL) relative to present. (A) Southeast Florida continental reef tract (light blue bars). (B) Florida Keys reef tract (purple bars). (C) Dry Tortugas coral-reef ecosystem (red bars). (D) All subregions combined (grey bars).

Our updated database of Holocene coral age-elevation data from the Florida reef tract is comprised of 303 total samples (Fig. 2; Stathakopoulos & Toth, 2019). The number of samples per reef subregion are as follows: 63 from the SFCRT, 149 from the FKRT, and 91 from the DTCRE. Of those samples, 87 are from *A*. *palmata*, 212 are from “massive” corals (this includes: *Colpophyllia natans*, *Diploria labyrinthiformis*, *Montastraea cavernosa*, *Orbicella* spp., *Porites astreoides*, *Pseudodiploria clivosa*, *Ps. strigosa*, *Stephanocoenia. intersepta*, and *Siderastrea siderea* samples), and four are from the branching coral *A*. *cervicornis*. The number of *A*. *palmata* samples from the SFCRT and the FKRT are nearly equivalent (41 and 46, respectively), whereas none were reported in studies through 2017 from the DTCRE. Recent observations confirm the rare presence of Holocene-aged *A. palmata* in the Dry Tortugas, however, and these ages could provide important sea-level data in the future (Toth et al., 2019). Details of the distribution of sample ages and elevations by reef subregion are shown in Figs. 2A–2C. When all samples are grouped together, they span an age range of 10.8 ka to present day and an estimated elevation range of +0.15 to −31.2 m MSL (Fig. 2D). In general, the FKRT and DTCRE possess relatively broader distributions of age and elevation ranges, whereas those for the SFCRT are concentrated within narrower intervals. This difference can be attributed to the earlier termination of reef accretion on the SFCRT (Stathakopoulos & Riegl, 2015) as well as the limited geospatial sampling of reef areas within that subregion (Fig. 1). Approximately 67% of all samples from the Florida reef tract have elevations from 2.5–12.5 m below MSL, and ∼60% of samples have ages within the 5–8 ka range. Clearly, the distribution of the ages and elevations of coral samples across the three reef subregions were affected by the selection of sampling sites by the original researchers.

**Figure 3:**
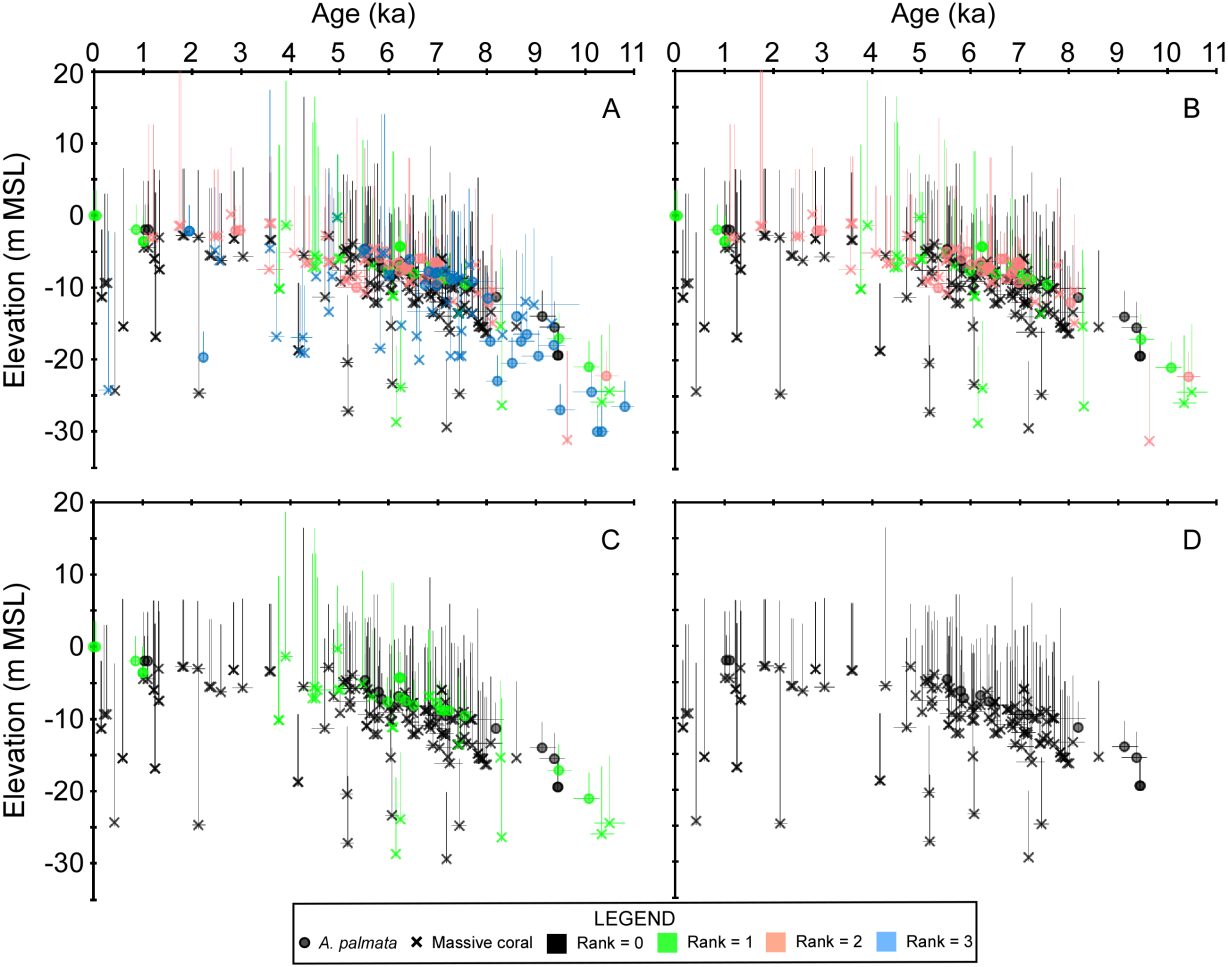
Plots of the coral age-elevation data iteratively removing samples based on the taphonomic screening criteria. Rankings range from the most stringent taphonomic criteria (rank = 0) to the most uncertain (rank = 3). Elevation is referenced to present mean sea level (MSL). Negative vertical error bars are the root sum square of the elevation uncertainties (2*σ*) and positive vertical error bars are the root sum square of the elevation uncertainties and the coral taxa depth distributions at 95% confidence intervals (2*σ*). Horizontal error bars (2*σ* uncertainty) are only depicted for samples that have age-error ranges ≥250 years. See Methods for explanations of error bars and screening procedures. (A) All coral data. (B) Coral data ranked from 0–2. (C) Coral data ranked from 0–1. (D) Coral data with a rank = 0 which were considered as in situ samples herein.

**Figure 4:**
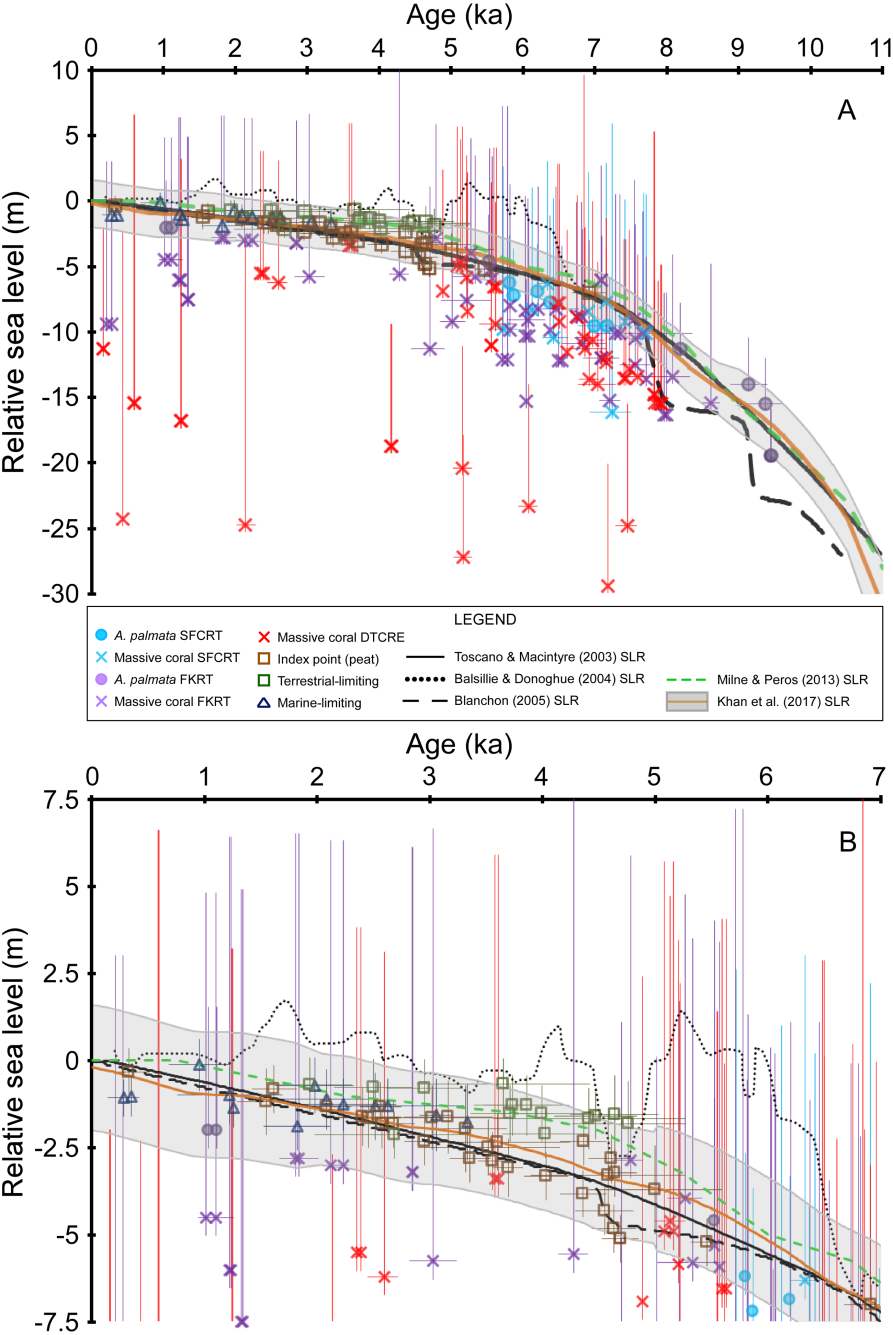
Sea-level data and sea-level reconstructions (SLRs) for south Florida. Coral age-elevation data from the Southeast Florida continental reef tract (SFCRT, light blue symbols), the Florida Keys reef tract (FKRT, purple symbols), and the Dry Tortugas coral-reef ecosystem (DTCRE, red symbols). Only coral data that passed our screening criteria employed in this study (i.e., rank = 0) are included. Negative vertical error bars are the root sum square of the elevation uncertainties (2*σ*) and positive vertical error bars are the root sum square of the elevation uncertainties and the coral taxa depth distributions at 95% confidence intervals (2*σ*). Horizontal error bars (2*σ* uncertainty) are only depicted for samples that have age-error ranges ≥250 years. Non-coral data (*brown*, *dark blue*, and *green symbols*) and their associated error bars are from Khan et al. (2017). See the Methods section for explanations of error bars and screening procedures. Relative sea level is referenced to present mean sea level. (A) Data for the entire Holocene record. (B) As in A, but only for the last 7 ka to depict the relationship between the non-coral database and the SLRs more clearly. The Blanchon (2005) SLR is a reinterpretation of the Toscano & Macintyre (2003) SLR.

Table 1 summarizes our analysis of taphonomic characteristics of the coral samples from our database. These criteria (which we used to determine whether a sample was in situ) indicate that of the samples which were available for analysis, ∼6% had basal attachments, ∼59% had normal corallite orientation, and ∼20% had normal geopetals. Of the 303 total coral samples, 69 were assigned a rank = 3 for either not passing the U-series or diagenetic screening criteria (34 samples, see Table S1 and Stathakopoulos & Toth, 2019) and/or for being unavailable for visual observation (42 samples). Fifty-nine samples were assigned a rank = 2 for not possessing any in situ indicators, being located within a sand interval (5 samples), and/or possessing significant age-reversals (5 samples), and 41 were assigned a rank = 1 for possessing ambiguous features. The remaining 134 samples were assigned a rank = 0 and are considered to have been collected in situ. Summary data plots of the screening procedure are shown in Fig. 3. All coral samples with a rank = 0 were plotted against the SLRs analyzed herein and are shown in Fig. 4. In addition, we plotted the non-coral samples reported in Appendix 3 of Khan et al. (2017), which includes 30 index points (from mangrove peats), and 13 marine-limiting and 14 terrestrial-limiting samples against the SLRs. These data span the age-range of ∼0.3–6.9 ka at elevations ranging from 0.1–7.0 m below MSL and help to better constrain the late Holocene portion of the coral database (see Figs. 2 and 4B). Together, the 191 coral and non-coral samples span a linear distance of nearly 450 km along the Florida reef tract and a few sites from the Florida Everglades region (Fig. 1). For comparison, the database used in the Khan et al. (2017) south Florida SLR was composed of 94 total samples and differs from our compilation only by the number of coral samples included (35 vs. 134 respectively). Our new database, therefore, provides a three-fold increase in the number of coral sea-level indicators available at present, all of which have been analyzed and taphonomically verified as in situ for the first time.

**Table 1:** Summary of the taphonomic characteristics of the 303 radiometrically dated coral samples from the south-Florida database. The table displays the number of observations for each taphonomic characteristic used to determine sample quality for suitability as sea-level data.

	Basal attachment	Normal corallite orientation	Normal geopetal
Yes	16	149	50
No	238	20	189
Indeterminate	3	85	14
Sample not available	46	49	50

### Subregional–regional trends and data gaps

Our new coral database highlights the availability and quality of sea-level indicators from the Florida reef tract across subregional to regional scales (Figs. 3 and 4). Although some gaps still exist in the age and elevation ranges of the coral data for individual reef subregions (Figs. 2A–2C), there is relatively good temporal coverage of combined coral and non-coral age-elevation data over most of the Holocene for the region (Figs. 2D and 4). The exception is during the early Holocene, as evidenced by the notable reduction of highly-ranked coral sea-level data before ∼8 ka (Figs. 3 and 4A; and described in greater detail in the ‘Discussion’ section). Of the 303 total coral samples from our south Florida database, we found that only 12% were from the early Holocene. Indeed, after applying our strict screening protocol and including only the highest-ranked coral samples (*N* = 134 for samples with a rank = 0), that number is reduced to 5%.

For most of the of the middle Holocene (∼8–5 ka), there is agreement (within uncertainties and <10 m between median depths) between most of the coral age-elevation data from at least two of the three subregions (Fig. 4A). Indeed, where *A. palmata* data are present within these intervals of high data density, the *A. palmata* largely plot among the shallowest massive coral data, which aids in constraining the upper uncertainty of deeper, coeval massive-coral data. The inclusion of the less-precise massive coral sea-level indicators (i.e., they possess relatively larger paleo water depths compared to the other indicators herein) in our database does result in some disagreements and offsets between the deepest and the shallowest coeval-coral data. For example, some massive corals from the DTCRE within the −15 to −30 m MSL-range plot outside of the uncertainty of shallower, coeval coral samples. The reason for the discrepancy of deep data could be that the samples were recovered from a deep coral reef on the Tortugas Bank (reef surface at −19 to −24 m MSL, Mallinson et al., 2003), which did not accrete on pace with sea level (Toth et al., 2018) and from the deeper forereef of Southeast Reef (reef surface at −15 m MSL, Shinn et al., 1977), demonstrating the variety of coral reef habitats encompassed in the coral database (see also Fig. 2). While these corals plot outside of their respective paleo water depth uncertainties when compared to other coeval corals (Fig. 4A), their paleodepths are still within their reported maximum depth ranges (Table 6 of Hibbert et al., 2018).

Overlap between the coral and non-coral datasets occurs during the period spanning the last ∼5.5 ka (prior to this period, there are only coral sea-level indicators and a single peat sample at ∼7 ka), which allows for comparisons between the various types of sea-level indicators during this time (Fig. 4). The terrestrial-limiting data, which span the last ∼5 ka, and the marine-limiting data which span the last ∼3.5 ka, agree (within error) with each other and with all other sea-level index points over this interval (Fig. 4B). From ∼5.5–4.3 ka, there is up to an ∼2 m offset between a few coeval peat sea-level index points; however, they are very close to, or within error of each other (Fig. 4B). This offset could be related to minor errors associated with the original determination of elevation or mangrove species, sediment compaction, and/or sample or radiocarbon contamination (Toscano & Macintyre, 2003; Toscano & Macintyre, 2005). Additional peat data from this timeframe would help to clarify which data are more reliable. The medians of the coral sea-level index points that overlap this interval fit better with the shallower trend of the peat dataset. Despite the relatively minor and short-lived inconsistency within the peat dataset, there is overall agreement between the terrestrial- and marine-limiting datasets (which help constrain the upper and lower positions of sea level, respectively) as well as with our coral dataset. The coherence of the non-coral and coral datasets suggests that a robust sea-level signal is likely captured by the two datasets during their period of overlap.

## Discussion

Our database provides the first comprehensive taphonomic characterization of the extensive archive of Holocene coral sea-level indicators from south Florida. Our study largely supports the conclusion by previous researchers that careful screening of samples is necessary to identify robust coral-based sea-level indicators (Blanchon & Perry, 2004; Blanchon, 2005) as more than half (∼56%) of the samples we evaluated could not definitively be determined to be in situ; however, we were able to identify 134 high-quality, coral samples in records from throughout the Florida reef tract, which represents a three-fold increase in the number of coral-based sea-level indicators available for developing SLRs compared to the most recently compiled sea-level database (Khan et al., 2017). Our database is therefore the largest compilation available at present and the only one that has been taphonomically verified.

### Sample characterization and data limitations

According to Blanchon & Perry (2004), basal-attachment surfaces provide the highest level of confidence for identifying in situ corals. The occurrence of basal attachments on samples from our database was very low: ∼6% of the radiometrically dated samples that were available for analysis definitively possessed a basal attachment (Table 1), and of these samples (*N* = 16), they were less common in *A. palmata* corals (19%) compared to massive corals (81%). However, a large proportion of these samples were originally collected and dated for studies intended to determine reef accretion histories rather than for the purposes of sea-level studies. Indeed, other coral specimens within many of the reef cores did possess basal contacts but they were not selected for radiometric dating by the original researchers (Stathakopoulos & Toth, 2019). An extensive study of 54 small-diameter cores from several eastern Caribbean sites by Hubbard, Burke & Gill (1998) also found little evidence of basal attachments and noted the difficulty in assessing whether corals were in situ using this criterion. It is possible that extensive bioerosion and alteration may be responsible for the lack of preserved basal attachments (cf. observations by Scoffin, 1972 from reefs in Bermuda). Whereas bioerosion and alteration were occasionally present in many of the cores from south Florida, we did not observe the persistent and highly altered scenarios described by Scoffin (1972) in our analysis. We did observe, however, that adjacent reef clasts within a core sometimes had rounded or smoothed-flat top/bottom surfaces which is clear evidence of the clasts grinding against each other during the drilling process. This phenomenon likely reduces the preservation potential of basal attachments. Based on our experience drilling and analyzing the cores from reef deposits in south Florida, we believe it is possible that basal attachments are rarely preserved in the Holocene record in general (e.g., Hubbard, Burke & Gill, 1998), or they become separated and destroyed due to the pressure and rotational forces exerted during core-drilling (see also Blanchon & Perry, 2004; Hubbard, 2011). It is also possible, however, that some of the corals were moved from their original growth position.

As a result of the low proportion of basal contacts in our samples, we largely relied on indicators other than basal contacts (i.e., normally oriented corallites and geopetals or combinations thereof). Of the samples that were available in our analysis, the dominant coral composition of their respective core intervals consisted of 64% massive corals, 18% mixed corals, and 17% *A. palmata*. Approximately ∼24% of these *A. palmata* samples were identified as *in situ* whereas ∼56% of massive corals from the massive and mixed-coral intervals were identified as in situ. The proportion of *in situ A. palmata* in our analysis is consistent with that found in other studies, suggesting that our screening protocols were robust. For example, Blanchon & Eisenhauer (2001) examined the Last Interglacial reefs from Barbados in outcrops and found that 17–30% of the *A. palmata* facies were comprised of in situ framework. Similarly, in their reef-coring study of Holocene reefs dominated by *A. palmata* (93% of core material) off the northwest Yucatan Peninsula, Blanchon & Perry (2004) reported that 25% of the recovered *A. palmata* was *in situ*. However, in a similar study off the northeast Yucatan Peninsula, Blanchon et al. (2017) reported that 90% of their recovered core material was comprised of *A. palmata*, of which only 10% was “rare in-place colonies of large *A. palmata* or head corals”. The relatively low proportion of in situ *A. palmata* in Holocene reef deposits has generally been attributed to storm-related transport, particularly on *Acropora*-dominated fringing reefs (Blanchon & Perry, 2004; Blanchon et al., 2017). Studies by Stoddart (1963), Woodley et al. (1981), and Scoffin (1993) demonstrated that branching corals, like acroporids, are much more susceptible to storm damage compared to massive corals, indicating that the latter are less likely to be transported and deposited as storm ridge accumulations (although smaller corals may be more susceptible to transport). For example, Williams et al. (1999) reported that subaerial ramparts adjacent to previously flourishing *A. palmata* reefs off southwestern Puerto Rico were composed of 88–98% of *A. palmata* clasts. We found, however, that the majority of all the Holocene reefs sampled from the Florida reef tract (∼30 sites) were primarily composed of massive corals (see also Toth et al., 2019), which are less likely to be dislodged and transported during storms. Of the samples that we identified as in situ, coeval *A. palmata* samples largely plot among the shallowest massive coral samples, thereby demonstrating a close coherence to the depth-related distributions between the two types of coral indicators and their robustness as recorders of sea-level (Fig. 4). Furthermore, Blanchon et al. (2017) noted that age-reversals between adjacent samples in their cores were diagnostic of storm-related deposition; however, despite the large number of samples dated in the cores <2% of all the samples from our database displayed significant age-reversals, which suggests that sampling of storm ridges or deposits was largely avoided (see also Toth et al., 2018; Toth, Stathakopoulos & Kuffner, 2018).

We emphasize that our interpretations of samples being in situ were based on several important assumptions: for example, we observed on several occasions that corallites of individual, massive coral skeletons were vertically oriented, but on a diagonal (i.e., subvertically). Some of these instances were clearly the result of the core-drill penetrating off the central axis of an individual coral head, and we designated these samples as having normal corallite orientation. However, this would allow the data to be included in some cases where coral samples were transported as allochthonous material and became emplaced within sections with their corallites still oriented vertically or subvertically (e.g., Lighty, Macintyre & Stuckenrath, 1982; Hubbard, Burke & Gill, 1998). It is possible that the high proportion of massive corals identified as in situ from our analyses may be an overestimate due to some of our designations for normally oriented corallites. It was also assumed that normal geopetals associated with coral samples were formed when the coral was in situ and that only inverted geopetals provide direct evidence of transported and redeposited material; however, Blanchon & Perry (2004) found both normal and inverted geopetals in the sections of their cores from the reef crest/flat and rubble cay zones. Although our database may include some coral samples that may not be in situ framework based on these assumptions, we believe the relatively large number of data points now available (e.g., Hubbard, 2011) after our careful analysis of the extensive USGS Core Archive nevertheless helps to further inform the general position of sea level throughout the south-Florida shelf. We reiterate that most of the age-elevation data from these cores were originally obtained for reef accretion studies and we note that future studies specifically interested in creating SLRs for the region should employ a hierarchical selection for dating coral samples that possess the most reliable taphonomic indicators. Samples with basal contacts should be prioritized, followed by samples with normally oriented corallites and geopetals.

### The early Holocene record

**Figure 5:**
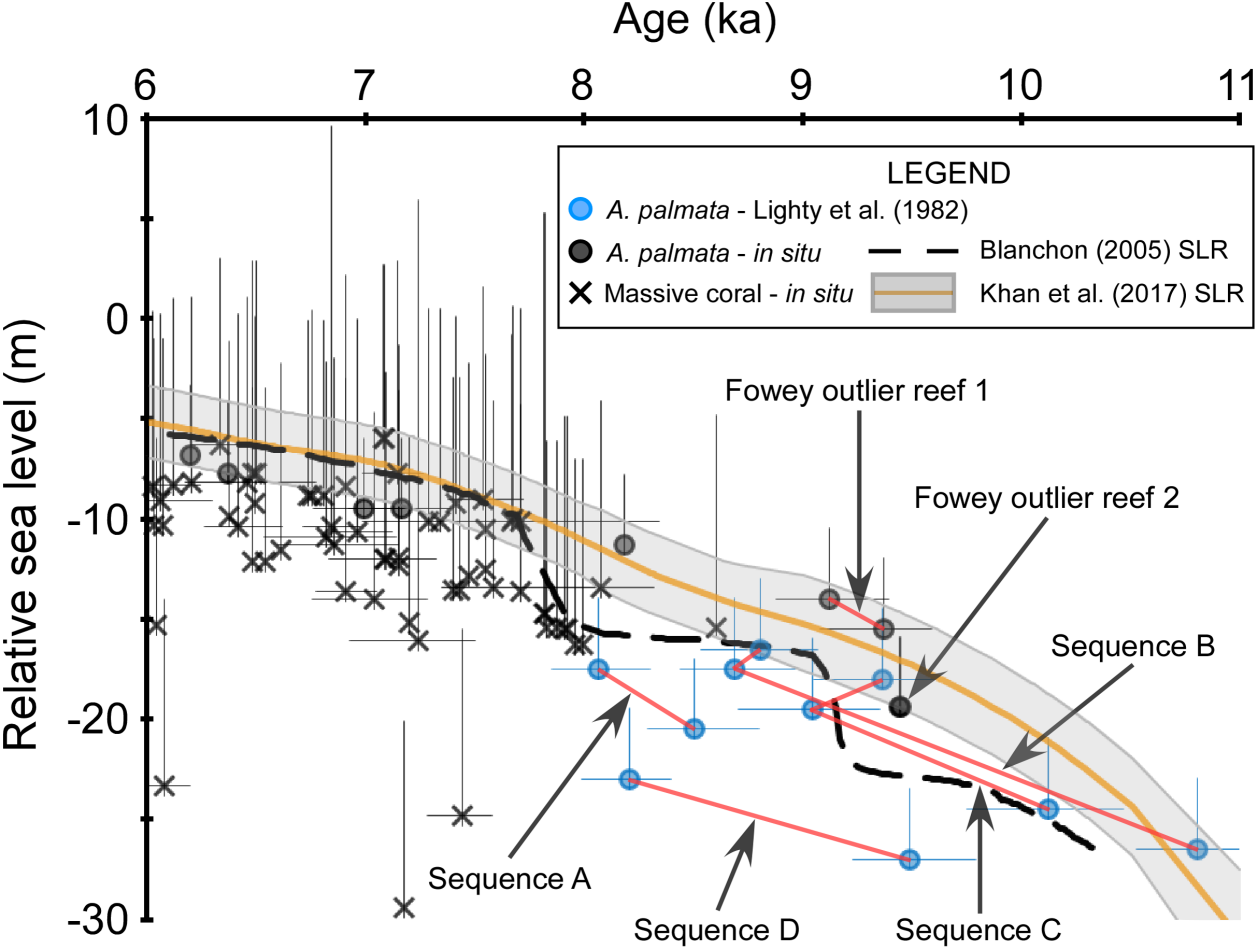
Discrepancies within the early Holocene sea level dataset. Coral data that passed our screening criteria are depicted as black symbols and are plotted with the data of Lighty, Macintyre & Stuckenrath (1982), blue symbols, which did not pass our screening because the associated samples were unavailable for visual analysis. *Red lines* connect samples that are from the same sampled outcrop (Sequences A–D from Lighty, Macintyre & Stuckenrath, 1982) or drill-core (Fowey outlier reef 1 from Lidz, Reich & Shinn, 2003). *Negative vertical error bars* are the root sum square of the elevation uncertainties (2*σ*) and positive vertical error bars are the root sum square of the elevation uncertainties and the coral taxa depth distributions at 95% confidence intervals (2*σ*). Horizontal error bars (2*σ* uncertainty) are only depicted for samples that have age-error ranges ≥250 years. See Methods for explanations of error bars and screening procedures. Relative sea level is referenced to present mean sea level. The Blanchon (2005) sea-level reconstruction (SLR) is a reinterpretation of the Toscano & Macintyre (2003) SLR.

The early Holocene observations recorded in previous south Florida sea-level databases and four of the five SLRs reviewed herein (all but the Balsillie & Donoghue, 2004 SLR) were based on age-elevation data from the reefs of the SFCRT initially studied by Lighty (1977) and Lighty, Macintyre & Stuckenrath (1978) and from deep, “outlier” reefs of the FKRT by Toscano & Lundberg (1998). Lighty (1977) examined a pipeline excavation through the “outer reef” (terminology after Banks et al., 2007) of the SFCRT and found that the reef was dominated by an *A. palmata* facies “with inclined, landward-oriented blades” (Lighty, 1977), indicating a shallow-water, in situ reef framework. These earlier data were used in the SLR by Lighty, Macintyre & Stuckenrath (1982) who stated that radiocarbon dates were taken from “in situ *A. palmata* coral samples…” and that “no samples were from storm-ridge deposits”. The context outlined above is important because it highlights that, despite efforts to only sample in situ reef-crest facies, there is variability in the *A. palmata* age-elevation data sampled from the four separate vertical transects within the outer reef at this location (Figs. 3 and 4 of Lighty, 1985; Fig. 5).

Whereas the age-elevation data from “Vertical Sequences B and C” (Table 2 of Lighty, Macintyre & Stuckenrath, 1982) demonstrate close coherence with one another, the data from “Vertical Sequences A and D” are offset from the former sequences in age and elevation, respectively (Fig. 5). Some of this variability may be explained by the relatively large age-uncertainties of these data; however, the lowermost age from “Vertical Sequence D” exceeds the originally assigned +5 m depth-range of monospecific *A. palmata* framework (offset up to ∼10 m) when compared with the immediately adjacent, younger data from “Vertical Sequences B and C”. Additional early Holocene data from the outlier reefs were included in the SLR by Toscano & Macintyre (2003) and were similarly offset by up to ∼12 m at ∼8 ka when compared to “Vertical Sequences A and D” (Fig. 4 of Toscano & Macintyre, 2003). Competing hypotheses for this seemingly large depth range of *A. palmata* framework, questioning of the reliability of these data, and the resulting SLR proposed in Toscano & Macintyre (2003) were highlighted in a series of comments and replies between Blanchon (2005) and Toscano & Macintyre (2005). A reinterpretation of the database used in the Toscano & Macintyre (2003) SLR led Blanchon (2005) to propose a stepped model of early Holocene sea-level rise (Fig. 1B of Blanchon, 2005) as a better fit for the data, which would also corroborate the previous hypothesis of Blanchon, Jones & Ford (2002) of early Holocene sea-level jumps. Although most recent SLRs from south Florida do not suggest any jumps in sea level occurred during this period (e.g., Milne & Peros, 2013; Khan et al., 2017), an abrupt jump in sea level during the early Holocene is supported by some studies from other locations. For example, studies from the Mississippi and Rhine-Meuse deltas using higher-resolution peat sea-level indicators have documented rapid jumps in local sea level at ∼8.5–8.2 ka that suggest periods of punctuated eustatic sea-level rise could have occurred during the early Holocene (Hijma & Cohen, 2010; Törnqvist & Hijma, 2012; Hijma & Cohen, 2019). Nevertheless, the variability of the data from south Florida used in these and subsequent analyses (see next section) is problematic and demonstrates how different interpretations of the same dataset have resulted in opposing conclusions.

Because our screening criteria excludes coral samples that were not available for visual characterization (see Methods), the data from the outer reef of the SFCRT, and thus, much of the early Holocene dataset that was used in the previous SLRs, are excluded from our comparisons with the five SLRs for south Florida (Fig. 3). Despite the large number of cores that were available for our analysis (Stathakopoulos & Toth, 2019) from locations throughout south Florida (Fig. 1), only six coral data points of early Holocene age passed our screening criteria (Fowey outlier reef, Marker G reef, Sand Key shelf-edge reef, and Sand Key outlier reef sites). We are therefore unable to critically evaluate the early Holocene portions of the SLRs herein (see next section) until additional, geospatially representative data are available; however, we compare the previous data to a newly-characterized, contemporaneous record from our study. Data from two cores from Fowey outlier reef (Lidz, Reich & Shinn, 2003; Toth et al., 2018; Toth, Stathakopoulos & Kuffner, 2018) indicate it is the only location sampled to date that is comparable to the outer reef of the SFCRT (locations “6” and “1” respectively from Fig. 1) in age-elevation range (∼4.7–10.5 ka compared to ∼8.0–10.6 ka) and the presence of thick (7.3–11.9 m compared to >10 m) monospecific sequences of *A. palmata*, some of which has distinct reef-crest signatures (Stathakopoulos & Toth, 2019). These data all plot well above the Blanchon (2005) SLR during the putative period of the early Holocene sea-level jumps (Fig. 4A) but they lack the resolution (sub-millennial) to entirely dismiss the possibility of sea-level jumps. Interestingly, these Fowey outlier reef age-elevation data, which were not included in any of the previous SLRs, agree better with the outer reef transects “Vertical Sequences B and C” that appear to have sampled the primary ‘core’ of the reef (Fig. 4 of Lighty, 1985; Fig. 5), and potentially indicates that the data from these transects are more reliable.

Accurately determining the rates and nature of early Holocene sea-level change is critical for SLRs in the region since the most significant sea-level rise occurred during this interval for most of the western Atlantic. The discrepancies associated with the early Holocene dataset, the disagreement among previous studies, and the lack of reliable data after our analysis demonstrates an obvious need for additional data from this period. Obtaining new early Holocene sea-level data from comparatively more precise peat sea-level indicators would be ideal; however, records are scarce for Florida (see Khan et al., 2017), and this period may therefore need to rely on a more data-dense compilation of coral indicators. Coral data from this period are relatively lacking for two reasons: (1) most early research focused on characterizing the shallow shelf-edge reefs of the FKRT (e.g., Shinn et al., 1977; Shinn, 1980), which primarily formed after ∼8 ka (Toth et al., 2018) rather than deeper offshore reef habitats, and (2) the increased logistical difficulty associated with diving and core-drilling to collect samples from reefs deeper than 10 m below MSL. The relative paucity of early Holocene sea-level data is a problem that is not unique to south Florida. For example, Khan et al. (2017) found that of the 737 sea-level data points from their study of 20 different locations throughout the Caribbean, only ∼10% were of early Holocene age. We suggest that future coral-sampling efforts could target reefs presently situated in deeper water (>10 m below MSL) that are known to be of early Holocene age, such as those along the extent of the outer reef of the SFCRT and the outlier reefs of the FKRT described above, to address this critical data gap.

### Relationship to Holocene sea-level reconstructions

The SLRs compared herein characterize three distinctly different trends of Holocene sea-level change for south Florida (Fig. 4). A generally smooth, monotonic rise to present sea-level is depicted in the SLRs of Toscano & Macintyre (2003), Milne & Peros (2013), and Khan et al. (2017), which are broadly similar because they relied on similar datasets, that we reiterate, were not taphonomically screened in detail for in situ characteristics prior to this study. These three SLRs differ by <2 m at most when compared to the mean of the Khan et al. (2017) SLR. Given that the 2*σ* error (grey-shading in Fig. 4) of the Khan et al. (2017) SLR (which is the only SLR to estimate uncertainty) captures all the variation among them, these SLRs will, therefore, be discussed together below. The Blanchon (2005) SLR, which is actually a reinterpretation of the data from Toscano & Macintyre (2003), depicts two early Holocene step-wise ‘jumps’ of ∼5 m in sea-level and a later, smaller jump of ∼1.5 m around ∼4.5 ka, but is otherwise identical to the Toscano & Macintyre (2003) SLR. The general agreement between these four SLRs over the last ∼7 ka is in stark contrast to the meter-scale highstand to lowstand oscillations in sea level depicted by the Balsillie & Donoghue (2004) SLR (Fig. 4B), which was based on geologic and archeological indicators (collected subaerially) from throughout the Gulf of Mexico continental margin and other global locations.

Our new database is the largest compilation of taphonomically characterized and quality-verified Holocene coral sea-level indicators from throughout the Florida reef tract available at present. We found that the potential discrepancies of early Holocene data from previous studies and the resulting lack of verified in situ coral samples from the early Holocene based on our screening procedure only allows us to confidently evaluate the last ∼7 ka at present. When our coral database is combined with the non-coral sea-level data and where there is sufficient data coverage, it provides a first-order test of the SLRs used in previous studies of the south-Florida region (Fig. 4). All new coral data included from our database agree with the Khan et al. (2017) SLR by either plotting below the median estimate of relative sea level or within their 2*σ* error-range (Fig. 4). The trends inferred from our database are still captured by their SLR, despite the fact that Khan et al. (2017) reconstruction did not incorporate many of the coral samples that have now been characterized in this study. For example, estimated sea level within a data gap centered at ∼6 ka in Fig. 7.10 of Khan et al. (2017) is still representative of the general trend implied by the densely populated data at that time in our Fig. 4. Our coral data from ∼7 ka to present also agree with the non-coral data that comprise this interval of the Khan et al. (2017) SLR.

Our data do not support the earlier hypothesis of meter-scale highstand to lowstand oscillations during the middle to late Holocene in south Florida as suggested by Brock et al. (2010) using the Gulf of Mexico SLR of Balsillie & Donoghue (2004) (Fig. 4B). From ∼6.5–5 ka, the Balsillie & Donoghue (2004) SLR plots too high in relation to the *A. palmata* and peat index points during this period. Some of the massive coral data plot just within error of this SLR, but most coral median depths are well below it. After ∼4.5 ka, nearly every terrestrial-limiting data point (which indicates subaerial elevation) would be submerged based on the Balsillie & Donoghue (2004) SLR, and it also submerges most of the peat data below their <0.6 m indicative range (based on modern tidal ranges reported for Florida, Toscano & Macintyre, 2003; Table 2 of Hibbert et al., 2016; Fig. 4 of Khan et al., 2017). Our coral data alone do not exclude the possibility of some smaller-scale (a few meters) or higher frequency variability in sea level, given the temporal and vertical resolution of this type of indicator; however, such oscillations are not apparent when combined with the non-coral data which have less vertical uncertainty (mean ± 0.8 m). Furthermore, studies by Törnqvist et al. (2004), Gonzalez & Törnqvist (2009), Milne & Peros (2013), and Khan et al. (2015) also found no evidence of sea-level oscillations or highstands with comparable magnitude or timing from the Gulf of Mexico. The smaller sea-level jump around ∼4.5 ka depicted in the Blanchon (2005) SLR occurs during an interval of coeval peat data that are offset in elevation; however, our coeval coral data agree better with the shallower peat trend, which suggest that no such jump occurred (Fig. 4B).

Recent studies and advances in SLRs (e.g., Toscano, Peltier & Drummond, 2011; Milne & Peros, 2013; Hijma et al., 2015; Khan et al., 2017 and references therein) demonstrate that incorporating data from various or far-removed regions to produce a relative SLR for a particular location is inappropriate due to differing isostatic responses, tectonics, and other local factors (see Shennan, 2015 and Horton et al., 2018 for a review). The older SLRs of Toscano & Macintyre (2003), Balsillie & Donoghue (2004), and Blanchon (2005) did not adhere to these guidelines partly due to the lack of robust, location-specific datasets and, therefore, relied on wider regional datasets out of necessity (Toscano & Macintyre, 2003; Toscano, Peltier & Drummond, 2011). The more recent SLRs by Milne & Peros (2013) and Khan et al. (2017) only incorporated the data of Toscano & Macintyre (2003) specifically from Florida, and further updated their SLRs by comparing them with geophysical models (Peltier, 2004; Peltier, Argus & Drummond, 2015), or some newer data and robust statistical models, respectively. Whereas the SLRs of Milne & Peros (2013) and Khan et al. (2017) demonstrated that site-specific variability in relative sea level exists throughout the western-Atlantic region, they also noted several limitations of the geophysical models, such as three-dimensional variations in mantle viscosity structure (e.g., Austermann et al., 2013) and the influence of tectonic processes. Furthermore, the incorporation of unscreened coral sea-level data from the Florida record likely reduces the precision of these SLRs, particularly during periods when coeval, non-coral sea-level indicators are not available. For example, during the early Holocene, the Toscano & Macintyre (2003), Milne & Peros (2013), and Khan et al. (2017) SLRs were primarily based on undifferentiated *A. palmata* data, which can possess vertical uncertainties of up to 10–15 m (compared to <5 m for in situ reef crest *A. palmata*), and could explain the >10 m offsets between coeval coral data (Fig. 5). Ongoing advances in the development of sophisticated geophysical models, estimates of uncertainties, and additional early Holocene data will aid in improving future SLRs for the Florida region.

## Conclusions

Our characterization of all previously dated coral samples and the resulting updated database presented here significantly improve understanding of Holocene coral-reef paleoecology and sea-level history in the south Florida region. Our 134 Holocene coral sea-level indicators from throughout the Florida reef tract represents a three-fold increase in the number of coral data points compared to the most recently constructed sea-level database (Khan et al., 2017), making it the largest compilation available at present and the only one that has been taphonomically verified. We show that the last ∼7 ka is well-constrained with sea-level data. Evaluation of the database against the most recent SLRs for south Florida largely supports the SLRs of Toscano & Macintyre (2003), Milne & Peros (2013), and Khan et al. (2017), which all suggest a monotonic rise over the last ∼7 ka; however, data comprising the early Holocene record are comparatively sparse, demonstrating that more data are required to further clarify this critical period. We identify locations from the Florida reef tract that contain early Holocene age sequences based on our analyses and suggest that future studies could target these sites for sampling to fill the gap in the record. We also suggest that future studies could employ a hierarchical selection of coral samples that possess the most reliable taphonomic indicators (i.e., preferentially target basal contacts first, then normally oriented corallites and geopetals) from reef sequences to provide the most accurate coral age-elevation data for SLRs in the future. Similarly, the collection of a more robust dataset of sea-level indicators from mangrove environments (most existing samples were collected prior to the advent of improved sampling, pre-treatment, and dating techniques specifically for peats) would help to better constrain the middle–late Holocene sea-level record. Improved SLRs could result from future sampling efforts using all types of sea-level indicators and targeting new and more geographically distributed locations within the reef subregions and throughout south Florida, which could help to elucidate whether subregional sea-level differences exist.

The broader uses of our database and our comprehensive approach to screening potential coral-based sea-level indicators include providing a framework that allows researchers to sort and screen samples with specific characteristics according to their desired study priorities (e.g., only reef crest *A. palmata* and massive corals with normally oriented corallites), and to similarly characterize and evaluate the quality of newly-collected samples. For example, some statistical sea-level models are more robust to a few potentially erroneous data points (Ashe et al., 2019), which could afford more liberal screening criteria. Our ranking system could also be used as a weighting system in such models, wherein the highest quality data are given more weight in the model while more uncertain data could be down-weighted. For more sensitive methods for reconstructing sea level, we suggest applying a conservative screening approach to characterize in situ corals and excluding any samples with potentially ambiguous characteristics (e.g., a coral with normally oriented corallites but also has an inverted geopetal). Detailed taphonomic characterizations from additional locations in the western Atlantic would also help to address the current knowledge gap regarding the preservation potential of in situ indicators in reef cores. Future research that aims to better understand Holocene sea-level history in the region could build upon this database and the criteria used herein to provide new high-quality sea-level data that can be incorporated into new models to improve the accuracy of SLRs.

##  Supplemental Information

10.7717/peerj.8350/supp-1 Table 1 Original and recalculated U-series data from referenced publications All data appear as they were originally reported. Data in *bold* text indicate recalculated values from this study. Data that are *underlined* failed the U-series screening criteria employed in this study. See Methods for further details.

## Additional Information and Declarations

### Data Availability

The following information was supplied regarding data availability: The sea-level database is available at: https://doi.org/10.5066/P98QFBJ3.
